# Effect of Water Vapor Generated by Fresh-Cut Mango (*Mangifera indica*) on the Release of β-Carotene from β-Cyclodextrin Inclusion Complexes Under Modified-Atmosphere Packaging

**DOI:** 10.3390/molecules31060976

**Published:** 2026-03-14

**Authors:** Andrés Leobardo Puebla-Duarte, Daniel Fernández-Quiroz, Ariadna Thalía Bernal-Mercado, Francisco Rodríguez-Félix, Rey David Iturralde-García, Miguel Ángel Robles-García, Saul Ruiz-Cruz, José de Jesús Ornelas-Paz, Ricardo Iván González-Vega, Carmen Lizette Del-Toro-Sánchez

**Affiliations:** 1 Departamento de Investigación y Posgrado en Alimentos, Universidad de Sonora, Blvd. Luis Encinas y Rosales S/N, Col. Centro, Hermosillo 83000, Sonora, Mexico; a215201577@unison.mx (A.L.P.-D.); thalia.bernal@unison.mx (A.T.B.-M.); francisco.rodriguezfelix@unison.mx (F.R.-F.); rey.iturralde@unison.mx (R.D.I.-G.); saul.ruizcruz@unison.mx (S.R.-C.); 2 Departamento de Ingeniería Química y Metalurgia, Universidad de Sonora, Blvd. Luis Encinas y Rosales S/N, Col. Centro, Hermosillo 83000, Sonora, Mexico; 3 Departamento de Ciencias Médicas y de la Vida, Centro Universitario de la Ciénega, Universidad de Guadalajara, Av. Universidad 1115, Col. Lindavista, Ocotlán 47820, Jalisco, Mexico; miguel.robles@academicos.udg.mx; 4 Centro de Investigación en Alimentación y Desarrollo A.C.-Unidad Cuauhtémoc, Av. Río Conchos S/N, Parque Industrial, Ciudad Cuauhtémoc 31570, Chihuahua, Mexico; jornelas@ciad.mx; 5 Departamento de Ciencias de la Salud, Centro Universitario de los Valles, Universidad de Guadalajara, Carr. A Guadalajara Km 45.5, Ameca 46600, Jalisco, Mexico; ricardo.gonzalez@academicos.udg.mx

**Keywords:** fresh-cut fruits, β-carotene, β-cyclodextrin, food packaging systems, storage stability, shelf-life extension

## Abstract

This study evaluated the effect of water vapor generated by fresh-cut mango (*Mangifera indica*) on the release of β-carotene from β-cyclodextrin complexes (β-C:β-CD) under stored Modified Atmosphere Packaging (MAP) and to demonstrate β-carotene stabilization and passive–active packaging behavior under MAP conditions. Containers with fresh-cut mangoes, with and without MAP (4% O_2_, 6% CO_2_, 90% N_2_), were prepared for monitoring over 6 days at 4 °C. β-C:β-CD complexes were incorporated into the lids of containers. The physicochemical, relative humidity, antioxidant, erythroprotective, microbiological, and biofunctional qualities of freshly cut mangoes during storage were analyzed. Active metabolic respiration of plant tissue led to a progressive decrease in O_2_ and an increase in CO_2_ in sealed containers, a phenomenon intensified by cutting, high humidity, and the system’s limited gas permeability. Application of MAP effectively modulated this microenvironment, reducing respiration rate, water loss, acidification, and the degradation of bioactive compounds. Compared to treatments without MAP, mangoes stored under modified atmosphere showed greater color stability, a slower rate of change in pH and titratable acidity, less loss of antioxidant activity and phenolic compounds, and significant preservation of erythroprotective capacity. Furthermore, MAP maintained microbial counts within the limits established by current regulations until the sixth day of storage. The encapsulation of β-C in β-CD effectively protected its bioactivity from oxidation, especially under MAP, although its release into the food matrix was limited, suggesting a predominantly passive behavior of the active packaging system. Overall, the results demonstrate that the combination of MAP constitutes a promising strategy for extending the shelf life and biofunctional stability of fresh-cut mangoes and β-C into the complex.

## 1. Introduction

Currently, fruits are in high demand among consumers due to their convenience as ready-to-eat products and various advantages, such as meeting their nutritional needs for vitamins, minerals, and antioxidants [1,2]. However, the fresh-cut processing of fruits accelerates physiological deterioration, leading to changes in texture, firmness, and color, while also increasing the risk of microbial spoilage and human pathogens due to tissue damage and biochemical changes, as well as an increase in respiration rate [3].

Mangos (*Mangifera indica* L.) are climacteric fruits known for their attractive flavor, aroma, and nutritional content [4]. However, mangos are highly perishable after harvest and are prone to fungal attack and accelerated respiration [5]. For this reason, it is necessary to store them in optimal conditions after post-harvest processing, also considering the packaging conditions during storage.

Food packaging plays a very important role in the food industry, as it is a strategic option for protecting and conserving food. It acts as a barrier to gases, water vapor, dust, and other external environmental agents. Packaging helps ensure the safety and quality of food products for consumers. However, there is a risk that the food has been contaminated during a process prior to packaging or that it has not been properly sterilized. Therefore, packaged foods are not immune to deterioration and pose a risk to the user’s or consumer’s health. In addition, the use of natural packaging solutions, such as biodegradable materials and modified atmosphere packaging (MAP), offers significant potential to reduce dependence on synthetic chemicals and enhance sustainability [6,7].

MAP is recognized as a sustainable technique with low energy consumption and CO_2_ emissions [8]. MAP combined with cooling processes is widely used in the food industry to address challenges with oxygen-sensitive foods [9]. This technique replaced the natural air concentration with a gas mixture, usually CO_2_ and N_2_, with lower O2 levels, to prolong shelf life by inhibiting the growth of microorganisms and preventing or slowing oxidation, especially of pigments such as carotenoids [10].

Carotenoids, such as β-carotene (β-C), are bioactive compounds that have attracted researchers’ attention because they can serve as antioxidants and natural colorants, and are extracted from abundant fruits and vegetables [11,12,13,14]. Carotenoids are known for their color changes (yellow, orange, and red); however, a disadvantage is that these bioactive compounds are hydrophobic pigments, susceptible to oxidation, and difficult to apply [15]. For that reason, the development of effective transport mechanisms for bioactive compounds, along with addressing the challenges posed by their natural properties, is of significant importance in modern food nutrition.

Incorporating inclusion complexes can effectively microencapsulate hydrophobic molecules such as β-C [16,17,18,19,20,21]. This approach is garnering attention in both the food and pharmaceutical industries due to its ability to enhance stability [7,11,22,23]. Cyclodextrin inclusion technology has recently shown promising results in enhancing the solubility, chemical stability, and bioavailability of bioactive compounds [20,24]. Moreover, cyclodextrins are considered non-toxic because they are biodegradable and biocompatible with the human body, which contributes to their Generally Recognized as Safe (GRAS) status.

Native cyclodextrins can be made up of 6 (α-cyclodextrin), 7 (β-cyclodextrin), or 8 (γ-cyclodextrin) glucose molecules, resulting in a cyclic oligosaccharide having the torus-shaped structure, creating a cone due to the units connected by α-1,4-glycosidic bonds [25]. When guest molecules are encapsulated within the hydrophobic cavities of cyclodextrins, this enhances the physical and chemical attributes of the guests through the formation of inclusion complexes [26,27]. Cyclodextrins improve shelf stability and simplify material handling. β-cyclodextrin (β-CD) is the most frequently utilized cyclodextrin due to its exceptional encapsulation capabilities [28]. It can effectively microencapsulate various hydrophobic molecules, including essential oils, lipids, carotenoids, vitamins, and similar compounds [29]. The outer surface of β -CD is polar; therefore, under humid conditions, water molecules can interact with the exterior, gradually releasing the entrapped nonpolar molecules. Therefore, this system enables the use of β-cyclodextrin inclusion systems for the sustained release of nonpolar compounds with potential applications in the preservation of fresh-cut fruits during storage.

Fruits, especially when minimally processed, such as freshly cut mango, begin to release moisture. This moisture accumulates within the packaging, resulting in a higher concentration over time. This accumulation can accelerate fruit deterioration by promoting microbial growth, oxidative reactions, and other spoilage processes. However, this situation, typically viewed as a disadvantage, can be leveraged as an advantage. The water vapor released by the fruit during storage can be utilized to adhere to the exterior of beta-cyclodextrin (on its polar side), facilitating the controlled release of encapsulated beta-carotene (which resides in the nonpolar interior). This gradual release of beta-carotene can then confer benefits to the fruit, potentially enhancing its preservation or nutritional value. Additionally, modified atmospheres, by reducing oxygen levels, could help reduce oxidation. However, no studies have been found on the effect that MAP could have on the release of B-C.

For this reason, this study aims to evaluate the effect of water vapor on fresh-cut mango (*Mangifera indica*) regarding the release of β-carotene from β-cyclodextrin complexes stored under modified atmosphere packaging (MAP), and to demonstrate β-carotene stabilization and passive–active packaging behavior under MAP conditions.

## 2. Results

### 2.1. Modified Atmosphere Packaging (MAP)

**Figure 1:**
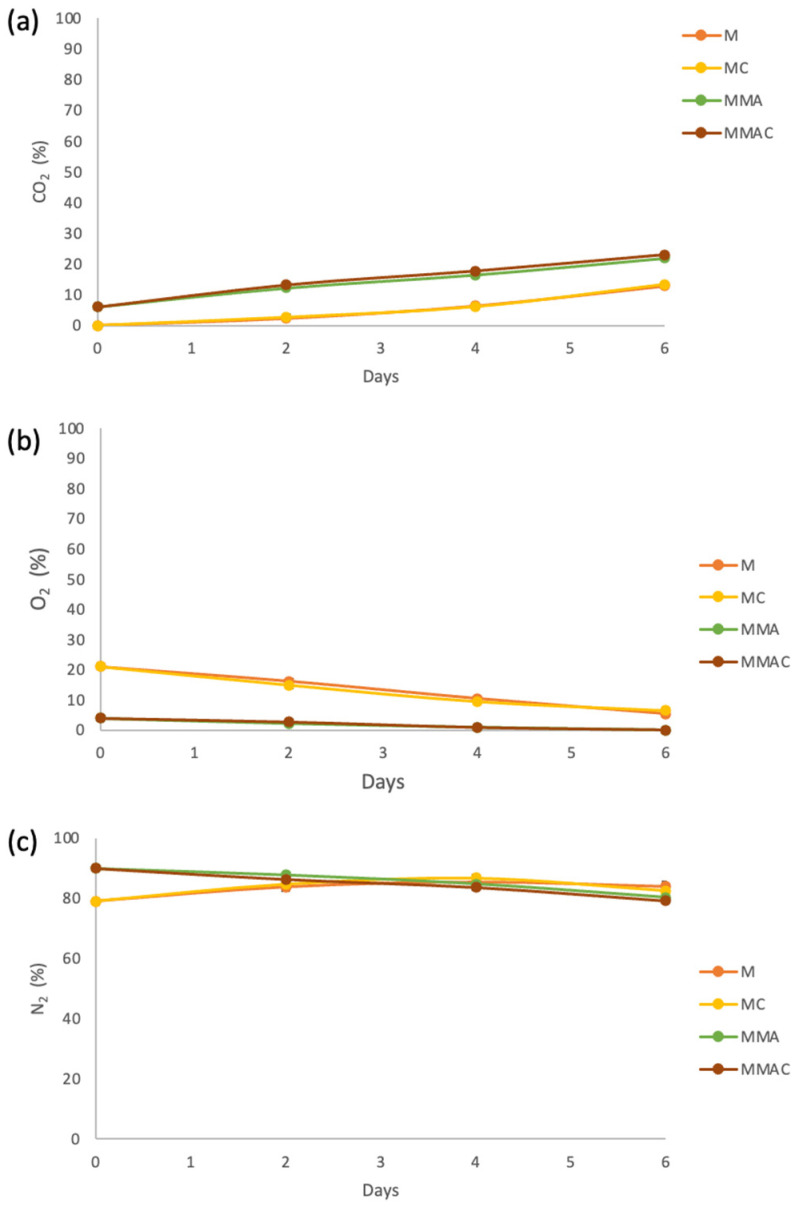
Variation of carbon dioxide (**a**), oxygen (**b**) and nitrogen (**c**) composition inside the package with freshly cut mango during 6 days of storage at 4 °C. M: Container of mango without MAP and without complex β-C:β-CD; MMA: Container of mango with MAP and without complex β-C:β-CD; MC: Container of mango without MAP and with complex β-C:β-CD; MMAC: Container of mango with MAP and with complex β-C:β-CD.

According to Figure 1, CO_2_ (Figure 1a) tended to increase and O_2_ (Figure 1b) to decrease in both samples with and without MAP. Nitrogen (Figure 1c) showed no significant difference between the samples treated with MAP and those that were not. O_2_ decreased more rapidly without MAP. The opposite occurred with CO_2_ content, which tended to increase slightly more rapidly under MAP conditions. The water vapor permeation coefficient in PET at 4 °C was 1.13 × 10^−14^ mol·m/(m^2^·s·Pa), indicating that gas permeability decreases significantly. The presence of inclusion complexes did not appear to have a significant effect on gas content or absorption, as no significant differences were observed between containers with and without the complexes.

### 2.2. Quantification of β-C from β-C:β-CD Complexes

Within the β-C:β-CD inclusion complexes, 3.298 ± 0.03 mg of β-C was initially retained and monitored during storage of fresh-cut mango with and without MAP (Table 1). Each day, a small amount of β-C was released from the β-C:β-CD complex, with the lowest release observed in the MAP-containing package (29%). Hence, the maximum amount of β-C released was 37% in the package without MAP. Therefore, MAP does affect β-C release, slowing it down.

### 2.3. Determination of Relative Humidity (RH)

**Figure 2:**
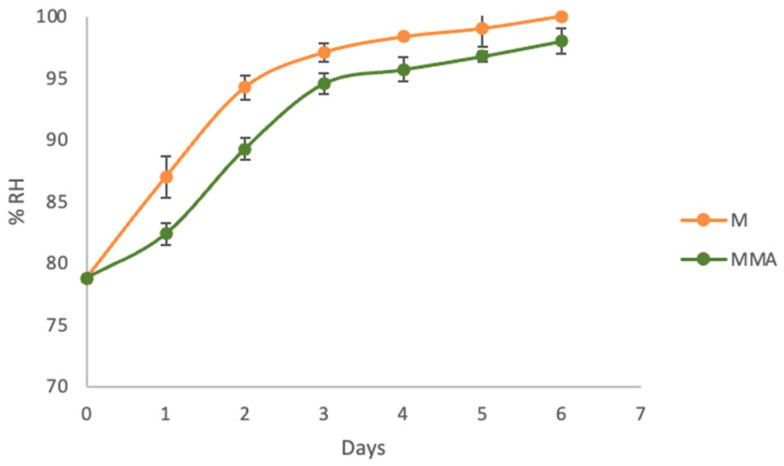
Variation in relative humidity (RH) inside the package with freshly cut mango during 6 days of storage at 4 °C. M: Container of mango without MAP; MMA: Container of mango with MAP.

Figure 2 shows the RH release kinetics. In both systems, RH increases during storage, but in the MAP container, it increases slightly more slowly. After 5 days, saturation is almost reached in the container with air. Over the first 3 days, there is approximately 3–6% less moisture release in an environment with MAP, and 3% by the 4th day. However, MAP does not prevent the increase in RH in sealed systems with freshly cut mango, but it modulates its kinetics, delaying the arrival of saturation compared to air packaging.

### 2.4. Color Determination

Table 2 presents the color results for mangoes during storage with and without MAP. The lightness (L*), a*, and b* parameters for mango decreased considerably in containers with air (M), and enzymatic browning was visible at 2 days of storage. However, in the MAP (MMA) packaging, L* also decreased slightly, but the change was visually imperceptible to the human eye. No drastic changes were detected in the values of a* and b*. The results showed that all MAP-related treatments, along with the inclusion complex, helped maintain the fruit skin color’s lightness compared to untreated controls.

Regarding the color of the complexes, remember that the samples were taken through filter paper bags. Generally, the complexed β-C appears as a soft red, but as storage days increase, the red color deepens, indicating that β-C is being released from the complex. However, it seems to remain in the filter paper bag rather than enter the headspace, which explains the increase in red color intensity in the complexes. However, in MAP (MMAC) containers, the color was less intense than in normal air (MC) containers.

### 2.5. Physicochemical Characterization of Fresh-Cut Mango

A kinetic study was carried out to determine the physicochemical changes presented by fresh mango cut at different times (0, 2, 4, and 6 days), when it is already fully ripe. Table 3 shows the physicochemical changes suffered by the mango in terms of the increase in total soluble solids (TSS, °Brix) and pH, as well as the decrease in the content of organic acids by the titratable acidity (TA) and firmness measure. Furthermore, the relationship between TSS and TA is expressed through the maturity index. In MAP containers, less mango deterioration, lower TSS, less increase in TA, and a slight decrease in pH and firmness were observed compared to samples in containers without MAP. M and MC samples showed similar behavior, with more deterioration of the mango. MMA and MMAC also showed no significant differences between the two treatments.

### 2.6. Total Phenols in Fresh-Cut Mango

**Figure 3:**
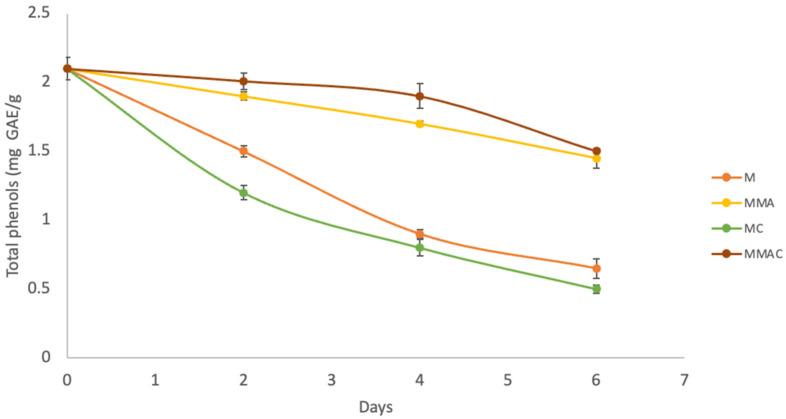
Determination of total phenols of fresh-cut mango stored at 4 °C during 6 days. Data are presented as mean ± SD (standard deviation) from at least three replicates (n ≥ 3). M: Container of mango without MAP and without complex β-C:β-CD; MMA: Container of mango with MAP and without complex β-C:β-CD; MC: Container of mango without MAP and with complex β-C:β-CD; MMAC: Container of mango with MAP and with complex β-C:β-CD.

Fresh-cut mangoes stored at 4 °C for 6 days (Figure 3) showed less degradation of phenolic compounds in samples preserved under MAP, with losses of 31% in MMA and 29% in MMAC at the end of the storage compared to the initial amount. These values indicate that, although phenolic compounds decrease progressively during storage, a considerable fraction remains stable under controlled atmosphere conditions. In contrast, the samples stored without MAP showed significantly greater degradation of phenolic compounds, reaching losses of 69% in M and 76% in MC at the end of the storage period. These results indicate greater susceptibility of phenolic compounds to degradation when fresh-cut mangoes are exposed to atmospheres with higher oxygen availability.

### 2.7. Antioxidant Activity

Table 4 presents the antioxidant activity of fresh-cut mango β-C from β-C:β-CD complexes stored at 4 °C in PET containers with and without MAP. According to the results, freshly cut mangoes stored in containers without MAP can lose approximately 45–58% of their antioxidant activity after 6 days of storage. Conversely, in MAP containers, this bioactivity is maintained for 2 days of storage, with only a slight loss of antioxidant activity after 6 days. The same behavior is observed with FRAP reducing power, which is maintained for a longer period in containers with MAP.

Regarding β-C from β-C:β-CD complexes, it was observed that, regardless of whether MAP is present, β-C maintains its antioxidant capacity, indicating that its encapsulation protects it from oxidation. It was also observed that it is better protected in environments with MAP due to its low oxygen content.

Furthermore, all freshly cut mango samples showed a greater affinity for the ABTS radical than for the DPPH radical. In contrast, the β-C of the β-C:β-CD complexes had a higher affinity for the DPPH radical, suggesting that more hydrophobic compounds have a higher affinity for the DPPH radical.

### 2.8. Erythroprotective Effect

**Figure 4:**
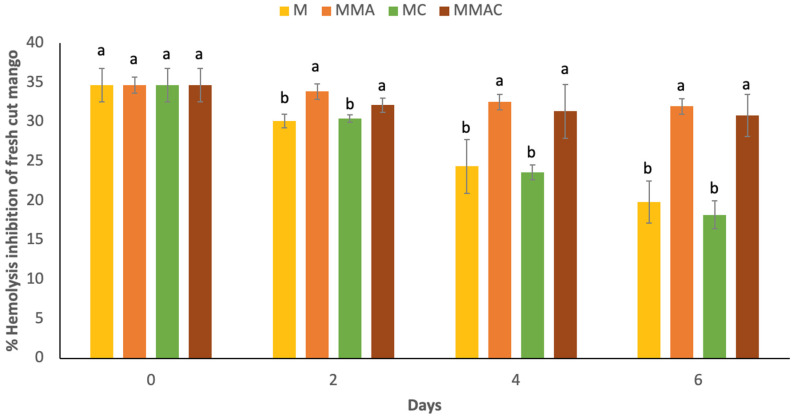
Hemolysis inhibition of fresh-cut mango extracts stored at 4 °C for 6 days. Data are presented as mean ± SD (standard deviation) from at least three replicates (n ≥ 3). Different lowercase letters represent significant sample differences for each storage day (*p* < 0.05). M: Container of mango without MAP and without complex β-C:β-CD; MMA: Container of mango with MAP and without complex β-C:β-CD; MC: Container of mango without MAP and with complex β-C:β-CD; MMAC: Container of mango with MAP and with complex β-C:β-CD.

**Figure 5:**
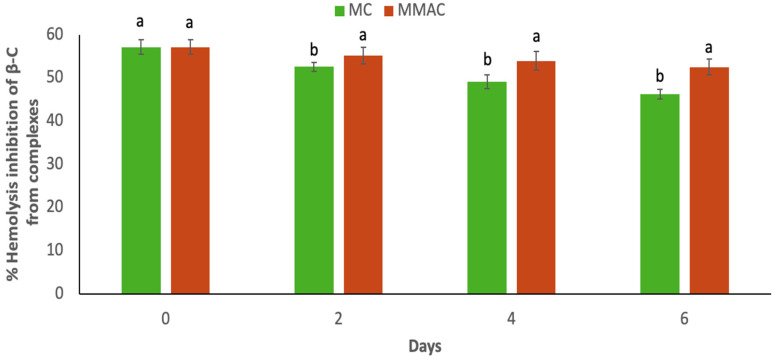
Hemolysis inhibition of β-C stored at 4 °C for 6 days. Data are presented as mean ± SD (standard deviation) from at least three replicates (n ≥ 3). Different lowercase letters represent significant sample differences for each storage day (*p* < 0.05). MC: Container of mango without MAP and with complex β-C:β-CD; MMAC: Container of mango with MAP and with complex β-C:β-CD.

Values of the protective effect of mango and β-C from complexes on human erythrocytes are presented in Figure 4 and Figure 5, respectively. In this assay, AAPH induces lysis of erythrocyte membranes due to the production of a peroxyl radical. If this radical is reduced by a proton donation (H+) from an antioxidant compound, hemolysis could be inhibited. Initially, mango showed hemolysis inhibition values of 34.70% (Figure 4). The decrease in erythroprotective capacity in containers without MAP is evident in fresh-cut mango. M and MC exhibit similar behavior. Even with the presence of the complex, MMA and MMAC show no significant differences, suggesting that β-C does not directly influence the erythroprotective capacity of mango. However, when this erythroprotection against β-C of the complexes was measured (Figure 5), it was observed that they have greater inhibition of hemolysis and that the complexes protect the bioactivity of this compound, since without MAP, it maintains 80% bioactivity and 92% with MAP after 6 days of storage.

### 2.9. Microbiological Analysis

Microbiological analyses (Table 5) indicate that deterioration of freshly cut mangoes becomes more visible after the fourth day in containers without MAP (M and MC), particularly due to the presence of mesophilic microorganisms. However, with MAP (MMA and MMAC), deterioration is slower, as mangoes can still be consumed after 6 days, within the limits established by the regulations (NOM-113, NOM-092, NOM-111).

## 3. Discussion

In sealed packages containing freshly cut mangoes, the decrease in O_2_ and the increase in CO_2_ are primarily due to the active metabolic respiration of the plant tissue. Once the fruit is cut from the plant, the synthesis of ATP and nutrient flow are disrupted, leading the mango to hydrolyze polymers to obtain high-molecular-weight compounds for respiration [30,31]. After cutting, the mango cells remain viable and continue to consume oxygen to oxidize respiratory substrates (mainly sugars), producing carbon dioxide as a byproduct [32,33]. The mechanical damage associated with peeling and chopping increases the respiration rate by exposing a larger cell surface area and activating stress-response metabolic pathways, thereby accelerating gas exchange [34,35,36]. Additionally, the sealed packaging limits O_2_ replenishment from the external atmosphere and promotes CO_2_ accumulation, creating an internal microenvironment with a modified gas composition. This effect is intensified by the high humidity and temperature of storage, as well as the absence of materials with high gas permeability [37]. Together, these factors explain the progressive shift in the internal atmosphere toward low O_2_ and high CO_2_ conditions, characteristic of minimally processed products.

Based on the above, decreased consumption of energy substrates, cell membrane degradation, and disorganization of cell walls in fresh-cut mangoes due to reduced oxygen levels result in better preservation of tissue integrity, thereby limiting water loss through cell collapse and exudation. This may explain why there is less RH in MAP containers compared to samples without MAP. Consequently, this affects the release of β-C from the complexes, since it is hypothesized that the binding of water molecules on the outside of β-CD could release the encapsulated material [38]. On the other hand, the water vapor permeation coefficient in PET at 4 °C was 1.13 × 10^−14^ mol·m/(m^2^·s·Pa), indicating that gas permeability decreases significantly in comparison with that at 23 °C (3.0 × 10^−14^ mol·m/(m^2^·s·Pa)); the latter was calculated for comparison purposes. Therefore, permeability decreases by approximately 60%, preventing more O_2_ from entering from the outside, less CO_2_ from exiting, and greater MAP stability during the 6 days.

Among the various characteristics of high-quality mangos, color is one of the most important factors as it influences consumer selection. All MAP-related treatments, along with the inclusion complex, helped maintain the fruit skin color’s lightness compared to untreated controls. This preservation effect is likely due to a synergistic delay in pigment degradation, attributed to reduced respiration rates and slower metabolic and enzymatic activity during storage. However, the red color of the complexes intensified under both normal conditions (MC) and with MAP (MMAC), because β-C is released from the complexes but apparently does not enter the headspace, thereby increasing the color from soft to stronger in the filter bag containing these complexes. Under MC conditions, the increase in red color was greater in the complexes within the filter paper bag, likely due to the higher relative humidity in these conditions compared with MMAC. The presence of moisture allowed for partial hydration of the β-cyclodextrin, which could induce decomplexation; however, the effective release of β-carotene was low, attributable to its highly hydrophobic and non-volatile nature, as well as the absence of a lipid phase to facilitate its diffusion or solubilization.

A series of biochemical reactions occurring within the fruit, which is reflected also in the loss of certain attributes, such as acids, and the gain of others, such as soluble solids and pH. TSS (°Brix), serves as a key indicator of a fruit’s nutritional quality and shelf life. The obtained values increased over time due to the hydrolysis of starches facilitated by the fruit’s own amylases, leading to the release of many glucose molecules and an increase in soluble solids [39,40]. This is reflected in the rise in °Brix, making the fruit sweeter.

On the other hand, during refrigerated storage of fresh-cut mangoes in PET containers, an inverse relationship between pH and titratable acidity is observed, a phenomenon widely reported in minimally processed fruits [40]. As storage progresses, pH tends to decrease, while titratable acidity increases, reflecting physio-logical and biochemical changes induced by cutting and packaging conditions. In the first few days of storage (days 0–2), the mango exhibits a relatively stable pH and low titratable acidity, characteristic of fresh tissue with active but controlled respiratory metabolism. However, mechanical damage caused by cutting increases cell permeability, facilitating the release of organic acids (mainly citric and malic acids) from the vacuole into the cytoplasm and the extracellular space, thereby contributing to the gradual increase in titratable acidity [41]. As storage progresses (days 3–6), the PET container’s oxygen permeability allows continued respiration. It promotes metabolic reactions associated with the degradation of sugars and organic acids, as well as the potential development of acidifying microflora (lactic acid bacteria and yeasts). These processes lead to a net accumulation of acids, evidenced by increases in titratable acidity and a progressive decrease in pH.

It is important to note that the pH does not decrease proportionally to the increase in titratable acidity, due to the mango’s buffering capacity, which is attributed to the presence of mineral salts, proteins, and natural acid-base systems in the plant tissue. For this reason, small pH variations can correspond to significant increases in titratable acidity, which explains why samples with similar pH values can show notable differences in their total acid content.

The use of MAP significantly slowed the decrease in pH and the increase in titratable acidity in fresh-cut mango. This behavior can be attributed to the modulation of respiratory metabolism and microbial activity induced by the gaseous composition of the environment. The reduction in available oxygen limits aerobic respiration in plant tissue, decreasing the rate of oxidation of substrates such as sugars and organic acids. On the other hand, the presence of 6% CO_2_ inhibits key metabolic enzymes and the growth of acidifying microorganisms, particularly lactic acid bacteria and yeasts, which are responsible for increased acidity during storage. The CO_2_ dissolved in the tissue’s aqueous phase forms carbonic acid in limited quantities; however, at the concentrations used, its net effect is not direct acidification but rather a reduction in metabolic and microbial activity, contributing to lower accumulation of organic acids.

It is important to note that, under MAP, the decoupling between pH and titratable acidity is less pronounced because the system maintains less disturbance of the tissue’s acid-base balance. This results in more gradual increases in titratable acidity and smoother pH variations, in contrast to packaging under normal atmospheric conditions, where the greater oxygen availability accelerates respiratory and fermentative processes. From a quality perspective, stabilizing pH and acidity under MAP conditions is associated with less development of acidic flavors, maintenance of sweetness, and delayed tissue softening. Consequently, an atmosphere containing 4% O_2_ and 6% CO_2_ effectively extends the shelf life of fresh-cut mangoes by mitigating the biochemical processes responsible for acidification.

The results also show that fresh-cut mango stored without a modified atmosphere (MAP) exhibited a considerable loss of antioxidant activity, with reductions of 45–58% after 6 days of storage. This can be attributed to the greater availability of oxygen, which favors oxidative processes and post-cut metabolism. In contrast, the use of MAP resulted in greater stability of antioxidant bioactivity, with values remaining practically unchanged during the first 2 days and losses of less than 20% at the end of storage. This effect is associated with reduced oxidative stress induced by low oxygen concentration and increased CO_2_, which limit the degradation of antioxidant compounds. Consistently, the reducing power determined by FRAP was better preserved in samples stored under MAP, indicating less oxidation of compounds with reducing capacity. Win and Setha [42] determined the antioxidant activity of mango samples treated with salicylic acid to preserve their quality and conserve their nutritional content, and found that untreated control samples decreased over time, losing antioxidant activity after 6 days, as in our results.

Various pigments, such as carotenoids, exhibit biological functions, including antioxidant activity [43,44]. Regarding β-C encapsulated in β-C:β-cyclodextrin complexes, its antioxidant capacity was largely maintained regardless of MAP use, demonstrating the protective effect of encapsulation against oxidation. However, the combination of encapsulation and MAP promoted greater stability of β-carotene, attributable to the low-oxygen environment. Additionally, samples of freshly cut mango showed a greater affinity for the ABTS•^+^ radical than for DPPH•, which is related to the mixed nature of the antioxidants present in the fruit matrix. In contrast, encapsulated β-carotene exhibited a greater affinity for the DPPH• radical, suggesting that more hydrophobic compounds interact preferentially with this radical, especially when encapsulation systems enhance their accessibility.

Polyphenols are recognized for their role in neutralizing free radicals through mechanisms such as hydrogen atom transfer (HAT), single-electron transfer (SET), and metal ion chelation, and are believed to play a major role in the health-promoting and pharmacological properties associated with mango consumption [45]. In this context, the use of MAP for storing fresh-cut mango significantly reduces the degradation of phenolic compounds (antioxidants) compared to storage without MAP, as evidenced by similar antioxidant activity. The lower losses observed in the MAP samples (31% in MMA and 29% in MMAC) suggest that reduced oxygen and increased CO_2_ in the storage environment limit the oxidative processes that degrade these compounds. In contrast, the higher losses recorded in the containers without MAP (69% in M and 76% in MC) can be attributed to greater oxygen exposure, which favors the oxidation of phenols and their participation in enzymatic and non-enzymatic reactions accelerated by tissue damage associated with cutting [3]. Overall, these results demonstrate that MAP effectively preserves phenolic compounds in fresh-cut mango, highlighting its potential to enhance the bioactive stability of the product during refrigerated storage.

On the other hand, the erythroprotective capacity of freshly cut mangoes decreases significantly during storage in containers without modified atmosphere packaging (MAP), with similar behavior observed between samples M and MC. This indicates that the presence of the β-carotene–β-cyclodextrin complex in the container lid does not directly influence the erythroprotection of the mango matrix. Consistently, the samples stored under MAP (MMA and MMAC) did not show significant differences between them, reinforcing the idea that the erythroprotective capacity of mangoes is primarily associated with their endogenous compounds and storage conditions, rather than a direct contribution from indirectly incorporated β-C. However, when erythro-protection was specifically evaluated for β-carotene from the complexes, greater inhibition of hemolysis was observed, demonstrating that encapsulation in β-CD protects the carotenoid’s bioactivity during storage. This effect was more pronounced under MAP, where β-C retained approximately 92% of its activity after 6 days, compared to 80% observed without MAP, suggesting an additional protective effect of the low-oxygen environment on the functional stability of the encapsulated compound.

The observed microbiological evolution indicates that the deterioration of fresh-cut mangoes accelerates from the fourth day of storage in the treatments without modified atmosphere (M and MC). This is mainly associated with an increase in mesophilic microorganisms, favored by high water activity, substrate availability, and oxygen exposure after minimal processing. In contrast, the modified atmosphere treatments (MMA and MMAC) showed slower deterioration kinetics, maintaining microbial counts within the limits established by Mexican Official Standards (NOM-113, NOM-092, and NOM-111) until the sixth day of storage. This effect can be attributed to the reduction in O_2_ and the increase in CO_2_ in the headspace, conditions that inhibit the respiratory metabolism of plant tissue and limit the growth of aerobic microorganisms, particularly mesophiles. These results confirm that the application of modified atmospheres is an effective strategy to prolong the microbiological shelf life of freshly cut mango, delaying deterioration and extending the safe consumption window of the product.

Finally, the complex’s location within the container lid limits direct contact with the food and reduces the concentration gradients required for significant migration, explaining the observed slow, partial release kinetics. In this context, the evaluated system is more suitable as passive–active packaging, whose main function is to act as an antioxidant reservoir and protective agent for the food’s environment, rather than as an active release system. These findings are consistent with previous reports indicating that cyclodextrins promote the stability of lipophilic compounds but limit their release, unless structural modifications or the incorporation of auxiliary systems are used [38]. Therefore, for applications requiring more efficient functional release, the use of hybrid systems or the integration of the complex into polymeric or lipid matrices is suggested, as these would improve the mobility and availability of β-carotene.

The methods applied differ in their assay principles and experimental conditions. Since multiple reaction characteristics and mechanisms are typically involved, no one assay can accurately reflect all antioxidants in a mixed or complex system. Therefore, to fully characterize the antioxidant capacity profile, different assays may be necessary.

## 4. Materials and Methods

### 4.1. Chemicals

β-Carotene, β-Cyclodextrin, ABTS [2,2-azino-bis-(3-ethylbenzothiazoline-6-sulfonic acid] powder, DPPH [2,2-diphenyl-1-picrylhydrazyl] powder, Trolox [6-hydroxy-2,5,7,8-tetramethylchroman-2- carboxylic acid] powder, TPTZ [2,4,6-tripridyl-s-triazine] powder, Tris-HCl biological buffer, AAPH [2,2-azobis(2-methylpropionamidine) dihydrochloride] powder or granules were purchased from Sigma-Aldrich (St. Louis, MO, USA). All other chemicals and solvents were of the highest commercial quality.

### 4.2. Materials

Mango (*Mangifera indica*) was purchased from a local supermarket (Sonora, Mexico) in November 2024 in a state of organoleptic maturity. The “Haden” mango variety was used in stage 4 (14–16 °Brix) of maturity, according to the Mango Handling and Ripening Protocol (https://www.mango.org/wp-content/uploads/2017/10/Mango_Handling_and_Ripening_Protocol_Eng.pdf, accessed on 6 November 2024), verifying that they did not present any damage.

### 4.3. Preparation of Mango

Mangoes were sanitized by immersion in chlorinated water (0.01% *v*/*v*), rinsed, and subsequently dried. To peel and cut the shell, a new material was used, which was also disinfected. Square samples (3 × 3 × 3 cm) of fresh-cut mangos (300 g) were placed in 0.5 L polyethylene terephthalate (PET) containers (11 × 11 × 7.5 cm). The containers were placed in a refrigerator at 4 °C for subsequent measurement for 6 days. All determinations were made in quadruplicate.

### 4.4. Preparation of Inclusion Complex

A precipitation technique was utilized, following the method described by Perez-Perez et al. [38] with modifications as described by Puebla-Duarte et al. [46]. Briefly, β-cyclodextrin (β-CD) was dissolved in a 1:2 ethanol:water mixture at 55 °C, while β-carotene (β-C) was dissolved in ethanol at a concentration of 10% (*w*/*v*). The β-C solution was gradually added to the β-CD solution to achieve a β-C:β-CD ratio of 40:60 (*w*/*w*). The mixture was stirred for 4 h at 25 °C in the absence of light, and subsequently stored at 4 °C for 12 h. The resulting precipitate was dried at 50 °C for 24 h.

The yield of the recovered inclusion complex, the entrapment efficiency, loading efficiency, UV–Visible, moisture content, release studios, adsorption–desorption isotherms, sorption kinetics, Fourier Transform Infrared Spectroscopy, Raman Spectroscopy, Differential Scanning Calorimetry, Thermogravimetric Analysis, X-Ray Diffraction, Zeta Potential, Scanning Electron Microscopy and Particle Size Distribution were reported by our working group in the publication of Puebla-Duarte et al. [46].

### 4.5. Modified Atmosphere Packaging (MAP)

Samples prepared as described in Section 4.3 were used, applying the methodology of Iturralde-García et al. [47] with a few modifications. The treatments were carried out by exposing the previously cut mangos to CO_2_, O_2_, and N_2_ (6, 4, and 90%) using a gas mixer (Thermco, La Porte, IN, USA). The normal atmosphere of the air was 21% O_2_; 0% CO_2_; 79% N_2_. An air inlet was placed through a hose; the PET lid displaced the air with MAP, and when the required concentration was reached, the lid was sealed. CO_2_, O_2,_ and N_2_ concentrations were monitored using a gas analyzer (Viasensor G110 CO2 Gas Analyzer, Viasensor, Didcot, Oxfordshire, UK) at both the commencement and conclusion of the treatment. Samples were monitored each day for 6 days.

Water vapor permeation coefficient (P(T)) in PET at 4 °C was calculated according to the Arrhenius model [48] according to Equation (1):

(1) $$P(4\;{}^{\circ}C)=P(T1)e^{-\frac{Ea}{R}(\frac{1}{T}-\frac{1}{T1})}$$

where P(T1) = permeability at 23 °C; T = 277 K (4 °C), T1 = 296 K (23 °C); Ea = activation energy for permeation (35 kJ/mol) and R = 8.3145 J/mol·K.

**Figure 6:**
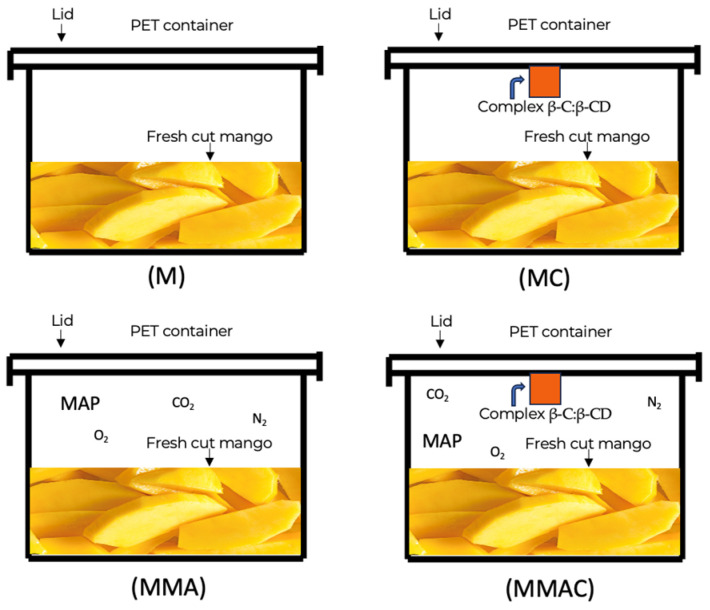
The different PET containers used in the study with freshly cut mangoes. M: Container of mango without MAP and without complex β-C:β-CD; MMA: Container of mango with MAP and without complex β-C:β-CD; MC: Container of mango without MAP and with complex β-C:β-CD; MMAC: Container of mango with MAP and with complex β-C:β-CD.

Additionally, in these same PET containers, filter paper bags (5 × 5 cm) containing 10 mg of the β-C:β-CD complex were placed on the inside of the lid, along with various controls, as shown in Figure 6.

### 4.6. Quantification of β-C in the Complex

During the storage of the freshly cut mango, the β-C content of the complexes was quantified. The β-C/β-CD inclusion complex (10 mg) was dissociated using 5 mL of ethanol and vortex-mixed for 3 min. The resulting dispersion was centrifuged at 4000 rpm for 5 min and then diluted 1:10 in the same solvent. The amount of β-C released into the organic phase was quantified using a calibration curve over 0–0.1 mg/mL. By measuring the β-C content within the complex, the release over time was estimated by difference [46].

All experiments were performed in triplicate. Quantification was carried out by UV–visible spectrophotometry (Multiskan Go, Thermo Scientific, Waltham, MA, USA) at 450 nm, using ethanol as the blank [46]. Additionally, the antioxidant capacity was quantified as described in Section 4.11 to assess the stability and bioactivity of the complexes.

### 4.7. Quantification of Relative Humidity in Packaging

The relative humidity (RH) inside the packages was determined using a precision digital hygrometer (Extech 445815 Hygro-Thermometer, Extech Instruments, Nashua, New Hampshire, USA), previously calibrated according to the manufacturer’s specifications. The hygrometer sensor was inserted into the container through an access port previously installed in the PET container wall. This port features a food-grade silicone septum that allows for leak-free insertion of the sensor. The silicone septum was pierced with the hygrometer probe, allowing the probe to be inserted directly into the headspace. The septum’s elasticity ensured automatic closure around the sensor, minimizing gas exchange with the outside. Measurements were recorded once internal equilibrium was reached. Readings were taken without opening the package, minimizing disturbances to the internal conditions. Measurements were performed throughout the storage period. Each determination was carried out in triplicate, and the results were expressed as a percentage of relative humidity (% RH). The data obtained enabled evaluation of moisture release dynamics and the microenvironment within packages containing freshly cut mango.

### 4.8. Color Determination

The color response of the β-C:β-CD inclusion complex and the mango fresh cut was determined. The color of both samples was measured at 5 random points using an automatic colorimeter (FRU WR10QC, Shenzhen Wave Optoelectronics Technology Co., Ltd., Shenzhen, Guangdong, China). The L* parameter indicates the lightness index on a scale from 0 (representing black) to 100 (representing white). The a* parameter quantifies the extent of redness (+a) or greenness (−a), while the b* parameter quantifies the extent of yellowness (+b) or blueness (−b). Determinations were performed at 4 °C, simulating the temperature commonly encountered during storage of fresh-cut fruit. Color changes were measured on days 0, 2, 4, and 6.

### 4.9. Physicochemical Characterization of Fresh-Cut Mango

Physicochemical assays were performed in triplicate according to the Official Methods of Analysis of the Association of Official Analytical Chemistry [49]. Total soluble solids (AOAC 932.12) were measured using a refractometer HI96813 (Hanna Instruments, Mexico City, Mexico) by placing a drop of mango juice on the refractometer, which was previously calibrated with distilled water, and reading the °Brix value. The pH (AOAC 981.12) was determined with a potentiometer (Ohaus Starter 5000 pH Meter, Ohaus, Parsippany, NJ, USA) by immersion of the electrode after calibration with buffer solutions. For titratable acidity (AOAC 942.15), 10 g of mango pulp were diluted in 50 mL of distilled water, filtered, and then titrated with 0.1 M NaOH. The results for titratable acidity were expressed as a percentage of citric acid. Firmness was determined according to Ayón-Reyna et al. [50]. It was measured in the central region of each sample using a Chatillon penetrometer (model DFE 100; AMETEK Inc., Largo, FL, USA) equipped with an 11 mm-diameter cylindrical probe. Measurements were taken at a penetration rate of 50 mm/min until a depth of 5 mm was reached. The values obtained were reported in Newtons (N).

### 4.10. Total Phenols of Fresh-Cut Mango

Total phenols were determined by the Folin–Ciocalteu method. Extracts were obtained by homogenizing 3 g of fresh-cut mangoes with 13 mL of methanol (80%). This preparation was sonicated (30 min) and centrifuged (4000 rpm, 15 min, 4 °C). The supernatant was filtered, and 10 µL was mixed with 25 μL of Folin–Ciocalteu (Sigma-Aldrich, St. Louis, MO, USA) (1 N) reagent for 5 min at room temperature. Subsequently, 25 μL of 20% Na_2_CO_3_ and 140 μL of distilled water were added to reach a final volume of 200 μL. The absorbance was measured at 760 nm after 30 min. Water was used as a blank (negative control). A curve was prepared using the gallic acid standard in methanol (0–600 μg/mL). The results were expressed as milligrams of Gallic Acid Equivalents per gram of dry weight (mg GAE/g DW). All measurements were performed in triplicate.

### 4.11. Antioxidant Activity

Antioxidant properties of fresh-cut mango and β-C from β-C:β-CD complexes were determined using the ABTS (2,2′-azinobis(3-ethylbenzothiazoline)-6-sulfonic) method [51], DPPH (2,2-diphenyl-1-picrylhydrazyl) method [52], FRAP (Ferric Reducing Antioxidant Power) method [53], and AAPH (2,2′-Azobis(2-amidinopropane) dihydrochloride) method [54]. For all tests, 1 g of a fresh-cut mango sample was used, ground, and subsequently diluted in 100 mL of ethanol. Extract of β-C from β-C:β-CD complexes was used as described in Section 2.6. Absorbance of the samples was measured with a UV-VIS microplate reader (Thermo Fisher Scientific Inc. Multiskan GO, Vantaa, Finland).

#### 4.11.1. ABTS

The radical ABTS method was implemented according to the procedure of Re et al. [51]. A solution of the radical ABTS·+ (0.0386 g/mL) was added with 88 µL of 2.45 mM potassium persulfate (K_2_S_2_O_8_) (Sigma-Aldrich, St. Louis, MO, USA). The mixture was left in a dark room for 12 h. Afterwards, 1 mL of this solution was collected and combined with the methanolic solution (CTR Scientific, Monterrey, Nuevo León, México) until an absorbance of 0.7 ± 0.02 at 734 nm was reached. The mango sample (20 μL) was combined with 270 μL of the radical solution and incubated in the dark for 30 min. The measurements were performed in triplicate. The results were expressed as micromoles of Trolox equivalents per gram of sample (µmol TE/g).

#### 4.11.2. DPPH

The antioxidant activity was assessed using a colorimetric method that measures the reduction in DPPH (a free radical) absorbance in the presence of the sample, as described by Loarca-Piña et al. [52]. The DPPH stock solution was diluted with ethanol (CTR Scientific, Monterrey, Nuevo León, México) to achieve an absorbance of 0.7 ± 0.02. Then, 200 µL of the DPPH solution was added to 20 µL of the mango extract, and the mixture was incubated in the dark for 30 min. Absorbance was recorded at 515 nm. The measurements were taken in triplicate, and the results were expressed as micromoles of Trolox equivalents per gram of sample (µmol TE/g).

#### 4.11.3. FRAP

FRAP was assessed by evaluating the reduction in ferric to ferrous ions following the method of Benzie and Strain [53]. First, individual sodium acetate buffer solutions (300 mmol/L, pH 3.6) were prepared along with an iron-TPTZ complex using FeCl3·6H2O (20 mmol/L) and a TPTZ solution in HCl (40 mmol/L). The FRAP reagent was then prepared by mixing the solutions in a 10:1:1 ratio (Buffer: FeCl_3_·6H_2_O: TPTZ·HCl). An aliquot of 280 µL of the FRAP reagent was combined with 20 µL of the sample, and the absorbance was measured at 638 nm. The measurements were conducted in triplicate, and the results were expressed as µmol TE/g sample based on a Trolox standard curve (4000 to 15.6 µmol).

### 4.12. Eritroprotective Effect

The assay was conducted individually for each sample using the technique described by González-Vega et al. [54] with AAPH. A blood sample was collected from a healthy volunteer aged 21 or older who had provided prior informed consent, in accordance with Mexican regulations (NOM-253-SSA1-2012). Approximately 5 mL of A+ blood was drawn into heparin tubes. A 2% erythrocyte solution was prepared by resuspending 1 mL of blood in 3 mL of physiological solution (PS, pH 7.4), homogenizing by inversion, and centrifuging at 2000 rpm for 10 min. This washing step was repeated three times. After removing the supernatant, 100 µL of the washed erythrocytes were suspended in 5 mL of physiological solution. The erythrocyte suspension was then mixed with each sample in Eppendorf tubes. AAPH (0.1085 g/mL, pH 7.4) was added as follows: samples (100 µL of erythrocyte suspension + 100 µL of AAPH + 100 µL of mango sample); positive control (C+) (100 µL of erythrocyte suspension + 100 µL of saline solution + 100 µL of AAPH); and negative control (100 µL of erythrocyte suspension + 200 µL of physiological solution). The tubes were incubated in a water bath at 37 °C for 3 h with constant shaking. After incubation, 1 mL of physiological solution was added to each tube, and the tubes were centrifuged at 2000 rpm for 10 min. Three hundred microliters of the supernatant were collected and transferred to a 96-well microplate. Absorbance (A) was measured at 540 nm. The results were reported as % inhibition of hemolysis according to Equation (2).

(2) $$\%\;Inhibition\;of\;hemolysis=\frac{\left(A_{C+}\right)-(A_{sample}-A_{PS})}{A_{C+}}\times 100$$

### 4.13. Microbiological Analysis

Fresh-cut mango (1 g) was sampled, diluted into 90 mL of peptone water, and mixed for 5 min. Evaluation of the viable microorganism content of mango FCF was carried out by quantifying total psychrophilic, mesophilic, fungi, and yeasts in Petri dishes by the plate counting technique. Consecutive decimal dilutions of the samples were prepared as stipulated in Official Mexican Standard NOM-110-SSA -1994, NOM-113-SSA, NOM-111-SSA, and NOM-092-SSA1-1994 [55,56,57,58]. Total coliforms, fungi, and yeasts were also quantified, and the results were compared with national and international reference microbiological criteria (total coliforms: ≤2.00 log_10_ CFU/g; mesophilic aerobic bacteria: ≤5.00 log_10_ CFU/g; fungi and yeasts: ≤3.00–4.00 log_10_ CFU/g). Serial dilutions were performed to measure microbial counts. After the incubation period, the colonies were counted and converted to the logarithm (base 10) of colony-forming units per gram of sample (log CFU/g).

### 4.14. Statistical Analysis

The experimental design was completely randomized. The data was analyzed using analysis of variance (ANOVA). The analysis of means was performed using Fisher’s least significant difference (LSD) test. Differences less than 0.05 (*p* < 0.05) were considered significant. Statgraphics Centurion XV (StatPoint Technologies Inc., Warrenton, VA, USA) software was used.

## 5. Conclusions

The results demonstrate that applying MAP (4% O_2_ and 6% CO_2_) to fresh-cut mango effectively modulates metabolic respiration and cutting-induced biochemical processes, resulting in a stable internal atmosphere that delays the physiological, microbiological, and oxidative degradation of the plant tissue. The reduction in available oxygen and the increase in CO_2_ limited the respiration rate, water release, acidification, and soluble solids loss, contributing to the maintenance of color, texture, and cell integrity during refrigerated storage. These conditions preserved the antioxidant activity, phenolic compounds, and erythroprotective capacity of the mango matrix, while also maintaining microbial counts within regulatory limits until the sixth day, effectively extending the product’s shelf life and safe consumption margin.

On the other hand, the encapsulation of β-carotene in complexes with β-cyclodextrin (β-C:β-CD) proved to be an effective strategy for preserving the carotenoid’s functional stability against oxidation, especially under MAP conditions. However, the release of the compound into the food matrix was limited by its hydrophobic nature and the complex’s location within the container lid. In this respect, the evaluated system primarily behaves as a passive–active packaging, acting as an antioxidant reservoir and modulating the food environment rather than as an active release system. Taken together, these findings highlight the potential of combining MAPs and encapsulation systems for the preservation of minimally processed fruits, while underscoring the need to develop hybrid matrices or additional structural strategies that enable more efficient functional release of lipophilic bioactive compounds.

## Figures and Tables

**Table 1:** Quantification of β-C from β-C:β-CD complex during storage in containers of fresh-cut mango with and without MAP.

	mg of β-C in the Complex β-C:β-CD							% β-C in the Complex	% β-C Released
Day	0	1	2	3	4	5	6		
MC	3.298 ± 0.03 ^Aa^	3.046 ± 2.23 ^Bb^	2.884 ± 3.65 ^Bc^	2.511 ± 1.87 ^Bd^	2.306 ± 2.09 ^Be^	2.219 ± 0.08 ^Bf^	2.072 ± 1.03 ^Bg^	62.8 ± 1.23 ^B^	37.1 ± 1.2 ^A^
MMAC	3.298 ± 0.03 ^Ba^	3.156 ± 1.42 ^Ab^	2.972 ± 1.19 ^Ac^	2.788 ± 0.71 ^Ad^	2.651 ± 2.04 ^Ae^	2.429 ± 0.88 ^Af^	2.336 ± 1.02 ^Ag^	70.8 ± 0.09 ^A^	29.2 ± 0.1 ^B^

Data are presented as mean ± SD (standard deviation) from at least three replicates (*n* ≥ 3). Different uppercase letters represent significant sample differences for each sample between columns (*p* < 0.05). Different lowercase letters indicate significant differences in bioassay results between rows (*p* < 0.05). MC: Container of mango without MAP and with complex β-C:β-CD; MMAC: Container of mango with MAP and with complex β-C:β-CD.

**Table 2:** Color parameters (L*, a*, and b*) of mango fruit and inclusion complex under MAP storage conditions (6% CO_2_, 4% O_2,_ and 90% N_2_; and Air conditions (0.03% CO_2_, 21% O_2_, and 78% N_2_).

Sample	Days	L*	a*	b*
Determination in the fresh-cut mango				
M	0	54.05 ± 0.89 ^a^	6.81 ± 0.19 ^a^	34.45 ± 1.42 ^a^
	2	43.16 ± 0.97 ^b^	3.86 ± 0.77 ^b^	20.66 ± 1.37 ^b^
	4	31.41 ± 0.89 ^c^	1.97 ± 0.04 ^c^	18.92 ± 1.58 ^c^
	6	22.50 ± 0.60 ^d^	0.78 ± 0.52 ^d^	16.32 ± 1.49 ^d^
MMA	0	54.05 ± 0.89 ^a^	6.81 ± 0.19 ^a^	34.45 ± 1.42 ^a^
	2	49.7 ± 1.65 ^b^	4.68 ± 1.38 ^b^	31.29 ± 2.31 ^b^
	4	47.52 ± 1.05 ^ab^	3.64 ± 0.38 ^bc^	27.76 ± 2.08 ^c^
	6	45.37 ± 0.69 ^c^	3.21 ± 0.74 ^c^	24.85 ± 1.09 ^d^
Determination in the β-C:β-CD complex				
MC	0	81.05 ± 1.04 ^a^	15.40 ± 0.41 ^d^	11.43 ± 0.34 ^d^
	2	82.53 ± 2.47 ^ab^	17.36 ± 1.73 ^c^	13.35 ± 0.63 ^c^
	4	83.47 ± 1.12 ^c^	18.21 ± 0.41 ^b^	15.21 ± 0.36 ^b^
	6	84.83 ± 0.59 ^cd^	21.33 ± 0.62 ^a^	19.23 ± 0.45 ^a^
MMAC	0	81.05 ± 1.04 ^a^	15.40 ± 0.51 ^bc^	11.43 ± 0.34 ^d^
	2	81.87 ± 1.03 ^a^	16.50 ± 1.42 ^b^	12.56 ± 0.36 ^c^
	4	82.34 ± 1.07 ^ab^	17.45 ± 1.57 ^ab^	13.84 ± 0.16 ^b^
	6	83.96 ± 1.02 ^bc^	18.48 ± 2.28 ^a^	16.12 ± 1.06 ^a^

Data are presented as mean ±SD (standard deviation) from at least six replicates (*n* ≥ 6). Different lowercase letters indicate significant differences between columns (*p* < 0.05). M: Container of mango without MAP and without complex β-C:β-CD; MMA: Container of mango with MAP and without complex β-C:β-CD; MC: Container of mango without MAP and with complex β-C:β-CD; MMAC: Container of mango with MAP and with complex β-C:β-CD.

**Table 3:** Physicochemical characterization of fresh-cut mango in PET containers at 4 °C storage during 6 days with and without MAP.

Sample	Days	TA %	TSS (°Brix)	pH	Firmness (N)
M	0	0.17 ± 0.001 ^d^	12.3 ± 0.09 ^d^	4.83 ± 0.02 ^a^	17.88 ± 1.32 ^a^
	2	0.32 ± 0.003 ^c^	14.1 ± 0.71 ^c^	4.72 ± 0.01 ^b^	14.03 ± 0.87 ^b^
	4	0.64 ± 0.009 ^b^	15.4 ± 1.01 ^bc^	4.67 ± 0.01 ^c^	11.33 ± 1.09 ^c^
	6	0.85 ± 0.060 ^a^	17.7 ± 2.35 ^a^	4.50 ± 0.09 ^d^	9.03 ± 0.23 ^d^
MMA	0	0.17 ± 0.001 ^c^	12.3 ± 0.09 ^c^	4.83 ± 0.08 ^a^	17.88 ± 1.32 ^a^
	2	0.21± 0.005 ^b^	13.4 ± 0.07 ^b^	4.83 ± 0.01 ^a^	16.98 ± 1.11 ^a^
	4	0.29 ± 0.003 ^b^	14.06 ± 1.01 ^ab^	4.81 ± 0.02 ^b^	16.03 ± 0.73 ^a^
	6	0.32 ± 0.009 ^a^	15.02 ± 1.14 ^a^	4.80 ± 0.02 ^b^	14.21 ± 1.07 ^b^
MC	0	0.17 ± 0.001 ^d^	12.3 ± 0.09 ^d^	4.83 ± 0.08 ^a^	17.88 ± 1.32 ^a^
	2	0.35 ± 0.007 ^c^	14.76 ± 1.41 ^bc^	4.76 ± 0.03 ^b^	13.07 ± 1.24 ^b^
	4	0.57 ± 0.002 ^b^	16.01 ± 2.31 ^ab^	4.67 ± 0.06 ^c^	10.33 ± 0.99 ^c^
	6	0.83 ± 0.008 ^a^	18.2 ± 2.18 ^a^	4.55 ± 0.01 ^d^	8.76 ± 0.52 ^d^
MMAC	0	0.17 ± 0.001 ^d^	12.3 ± 0.09 ^c^	4.83 ± 0.08 ^a^	17.88 ± 1.32 ^a^
	2	0.20 ± 0.019 ^c^	13.88 ± 0.99 ^b^	4.80 ± 0.02 ^a^	17.32 ± 2.01 ^a^
	4	0.27 ± 0.021 ^b^	14.01 ± 1.57 ^ab^	4.80 ± 0.01 ^a^	16.86 ± 1.06 ^a^
	6	0.31 ± 0.002 ^a^	14.08 ± 1.33 ^a^	4.78 ± 0.06 ^b^	15.34 ± 0.97 ^ab^

Data are presented as mean ±SD (standard deviation) from at least three replicates (*n* ≥ 3). For firmness, there were nine replicates. Different lowercase letters indicate significant differences between columns (*p* < 0.05). M: Container of mango without MAP and without complex β-C:β-CD; MMA: Container of mango with MAP and without complex β-C:β-CD; MC: Container of mango without MAP and with complex β-C:β-CD; MMAC: Container of mango with MAP and with complex β-C:β-CD. TSS: Total Soluble Solids; TA: Titratable acidity.

**Table 4:** Antioxidant activity of fresh-cut mango and β-C from β-C:β-CD complexes stored at 4 °C in PET containers with and without MAP.

Sample	Day	ABTS (%)	DPPH (%)	FRAP (mMol ET/mL)
Determination in the fresh-cut mango				
M	0	35.17 ± 2.67 ^a^	11.29 ± 3.51 ^a^	6353.66 ± 346.59 ^a^
	2	28.55 ± 0.93 ^b^	9.38 ± 3.09 ^ab^	5845.67 ± 413.55 ^b^
	4	19.31 ± 1.48 ^c^	7.31 ± 1.78 ^b^	4259.52 ± 394.40 ^c^
	6	14.72 ± 3.22 ^d^	4.73 ± 1.25 ^c^	3360.75 ± 846.56 ^d^
MMA	0	35.17 ± 2.67 ^a^	11.29 ± 3.51 ^a^	6353.66 ± 346.59 ^a^
	2	35.55 ± 1.42 ^a^	10.99 ± 1.01 ^a^	6154.97 ± 301.06 ^a^
	4	32.22 ± 2.08 ^b^	9.71 ± 0.32 ^ab^	6033.12 ± 104.55 ^a^
	6	28.09 ± 1.70 ^c^	8.20 ± 0.81 ^b^	4896.19 ± 279.12 ^b^
MC	0	35.17 ± 2.67 ^a^	11.29 ± 3.51 ^a^	6353.66 ± 346.59 ^a^
	2	29.21 ± 1.74 ^b^	10.32 ± 1.11 ^a^	5723.51 ± 205.11 ^b^
	4	21.32 ± 0.99 ^c^	8.21 ± 1.04 ^b^	4356.98 ± 173.02 ^c^
	6	15.76 ± 0.45 ^d^	3.94 ± 0.85 ^c^	3587.05 ± 345.66 ^d^
MMAC	0	35.17 ± 2.67 ^a^	11.29 ± 3.51 ^a^	6353.66 ± 346.59 ^a^
	2	34.99 ± 1.03 ^a^	11.03 ± 1.07 ^a^	5965.55 ± 54.23 ^a^
	4	31.53 ± 2.51 ^b^	9.55 ± 0.88 ^b^	5702.88 ± 112.76 ^b^
	6	26.87 ± 2.09 ^c^	7.76 ± 0.97 ^c^	4003.19 ± 108.45 ^c^
	Determination in the β-C from β-C:β-CD complex			
MC	0	45.22 ± 3.09 ^a^	57.87 ± 2.54 ^a^	25.32 ± 1.43 ^a^
	2	40.15 ± 1.08 ^b^	53.65 ± 1.17 ^b^	23.21 ± 1.11 ^b^
	4	38.33 ± 1.01 ^c^	49.01 ± 2.22 ^c^	21.45 ± 2.02 ^c^
	6	36.98 ± 1.31 ^d^	45.87 ± 1.33 ^d^	19.97 ± 1.36 ^d^
MMAC	0	45.22 ± 3.09 ^a^	57.87 ± 2.54 ^a^	25.32 ± 1.43 ^a^
	2	44.76 ± 1.32 ^a^	57.44 ± 1.38 ^a^	25.89 ± 2.08 ^a^
	4	42.22 ± 2.04 ^b^	56.93 ± 1.04 ^a^	23.43 ± 1.22 ^a^
	6	41.77 ± 1.81 ^b^	53.75 ± 1.51 ^b^	21.09 ± 1.43 ^b^

Data are presented as mean ±SD (standard deviation) from at least three replicates (*n* ≥ 3). Controls in ABTS, DPPH, and FRAP determinations were prepared with the solvent used for the samples. Different lowercase letters indicate significant differences between columns (*p* < 0.05). M: Container of mango without MAP and without complex β-C:β-CD; MMA: Container of mango with MAP and without complex β-C:β-CD; MC: Container of mango without MAP and with complex β-C:β-CD; MMAC: Container of mango with MAP and with complex β-C:β-CD.

**Table 5:** Microbial counts expressed as log_10_ (CFU/g) of fresh-cut mango stored at 4 °C with and without MAP.

	Day	Mesophilic	Psychrophilic	Fungi/Yeasts	Coliforms
M	0	<2.40	<2.40	<2.00	<1.00
	2	3.16	<2.40	<2.40	<1.00
	4	5.60	<2.40	4.69	<1.00
	6	6.10	<2.40	5.23	<1.00
MMA	0	<2.40	<2.40	<1.00	<1.00
	2	<2.40	<2.40	<1.00	<1.00
	4	2.94	<2.40	<2.40	<1.00
	6	3.08	<2.40	3.15	<1.00
MC	0	<2.40	<2.40	<1.00	<1.00
	2	3.16	<2.40	<2.40	<1.00
	4	5.61	<2.40	4.57	<1.00
	6	6.06	<2.40	5.20	<1.00
MMAC	0	<2.40	<2.40	<2.00	<1.00
	2	<2.40	<2.40	<2.00	<1.00
	4	2.99	<2.40	<2.40	<1.00
	6	3.15	<2.40	3.10	<1.00

Values are expressed as log_10_ (CFU/g).

## Data Availability

The original contributions presented in this study are included in the article. Further inquiries can be directed to the corresponding author(s).

## Abbreviations

The following abbreviations are used in this manuscript:

MAP	Modified Atmosphere Packaging
β-C	β-carotene
GRAS	Generally Recognized as Safe
β-CD	β -cyclodextrin
PET	Polyethylene terephthalate
RH	Relative humidity
M	Sample of mango without MAP and without complex
MC	Sample of mango without MAP and with complex β-C:β-CD
MMA	Sample of mango with MAP and without complex β-C:β-CD
MMAC	Sample of mango with MAP and with complex β-C:β-CD
AOAC	Association of Official Analytical Chemistry
ABTS	2,2′-azinobis(3-ethylbenzothiazoline)-6-sulfonic
DPPH	2,2-diphenyl-1-picrylhydrazyl
FRAP	Ferric Reducing Antioxidant Power
Trolox	6-hydroxy -2,5,7,8-tetramethylchroman-2-carboxylic acid
TPTZ	2,4,6-Tripridil-s-triazine
AAPH	2,2′-Azobis(2-amidinopropane) dihydrochloride
PS	Physiological solution
A	Absorbance
C+	Positive control
rpm	Revolutions Per Minute
TSS	total soluble solids
TA	Titratable acidity
HAT	Hydrogen atom transfer
SET	Single electron transfer
